# Neutrophil, lymphocyte and platelet ratio as a predictor of postoperative acute kidney injury in major abdominal surgery

**DOI:** 10.1186/s12882-018-1073-4

**Published:** 2018-11-12

**Authors:** Joana Gameiro, José Agapito Fonseca, Joana Monteiro Dias, Joana Milho, Rosário Rosa, Sofia Jorge, José António Lopes

**Affiliations:** 1 Division of Nephrology and Renal Transplantation, Department of Medicine, Centro Hospitalar Lisboa Norte, EPE, Av. Prof. Egas Moniz, 1649-035 Lisbon, Portugal; 2 Department of Surgery, Centro Hospitalar Lisboa Norte, EPE, Av. Prof. Egas Moniz, 1649-035 Lisbon, Portugal

**Keywords:** Acute kidney injury, Inflammation, Neutrophil, Lymphocyte, Platelets

## Abstract

**Background:**

Surgery is one of the leading causes of acute kidney injury (AKI) in hospitalized patients. Major abdominal surgery has the second higher incidences of AKI, after cardiac surgery. AKI results from a complex interaction between hemodynamic, toxic and inflammatory factors. The pathogenesis of AKI following major abdominal surgery is distinct from cardiac and vascular surgery. The neutrophil, lymphocytes and platelets (N/LP) ratio has been demonstrated as an inflammatory marker and an independent predictor for AKI and mortality after cardiovascular surgery. The aim of this study was to evaluate the prognostic ability of the post-operative N/LP ratio after major abdominal surgery.

**Methods:**

We cross-examined data of a retrospective analysis of 450 patients who underwent elective or urgent major nonvascular abdominal surgery at the Department of Surgery II of Centro Hospitalar Lisboa Norte from January 2010 to February 2011. N/LP ratio was determined using maximal neutrophil counts and minimal lymphocyte and platelet counts in the first 12 h after surgery. AKI was considered when developed within 48 h after surgery.

**Results:**

One-hundred and one patients (22.4%) developed AKI. Patients with higher N/LP ratio had an increased risk of developing postoperative AKI (6.36 ± 7.34 vs 4.33 ± 3.36, *p* < 0.001; unadjusted OR 1.1 (95% CI 1.04–1.16), *p* = 0.001; adjusted OR 1.05 (95% CI 1.00–1.10), *p* = 0.048). Twenty-nine patients died (6.44%). AKI was an independent predictor of mortality (20.8 vs 2.3%, *p* < 0.0001; unadjusted OR 11.2, 95% CI 4. 8-26.2, *p* < 0.0001; adjusted OR 3.56, 95% CI 1.0 2-12.43, *p* = 0.046). In a multivariate analysis higher N/LP ratio was not associated with increased in-hospital mortality.

**Conclusion:**

Postoperative N/LP ratio was independently associated with AKI after major abdominal surgery, although there was no association with in-hospital mortality.

## Background

Postoperative acute kidney injury (AKI) accounts for up to 40% AKI cases in hospitalized patients [1] and has been associated with progression to chronic kidney disease (CKD), increased cardiovascular events, increased length of hospital stays and increased in-hospital and long-term mortality [2–5].

The incidence of AKI in surgical patients is variable depending on the surgical setting, with higher rates being reported after cardiac, general, and thoracic surgeries. [5, 6] Nevertheless, the pathogenesis of AKI following major abdominal surgery appears to be distinct from that of cardiac and vascular surgery.

In fact, AKI after major abdominal surgery is multifactorial and results from an intricate interaction of hemodynamic, toxic and inflammatory factors. [7, 8] Also, the role of a pro-inflammatory response after abdominal surgery resulting from the post-ischemic or reperfusion period has been increasingly recognized in AKI [9–12] and appears to negatively impact other organs. [9].

Given the short and long-term impact of post-operative AKI, it is highly important to detect predictors and early markers of AKI in this setting in order to timely prevent and manage this complication. [8].

The neutrophil-lymphocyte ratio (N/L ratio) is a low-cost biomarker of systemic inflammation, easily calculated from a complete blood cell count. [13].

Its role has a prediction tool in cardiovascular mortality [14], survival in malignancies [15], postoperative outcome [16], and progression of CKD [17] has been previously demonstrated. Furthermore, in recent studies it has been studied as an early predictor for AKI in the emergency setting [13], in septic patients [18], contrast induced-AKI [19] and in cardiovascular surgery [20]. In cardiovascular surgery it was also associated with one-year mortality [20]. More recently, the addition of platelet count to this ratio increased predictive ability of AKI compared to N/L ratio or platelet nadir after cardiovascular surgery [21].

However, the prognostic ability of the neutrophils to lymphocytes and platelets ratio (N/LP ratio) has not previously been evaluated in AKI after major abdominal surgery.

The aim of this study was to evaluate the prognostic ability of the post-operative N/LP ratio after major abdominal surgery. For this purpose, we cross-examined data from a retrospective study in which we studied a cohort of patients undergoing major nonvascular abdominal surgery in which the objective was to evaluate the incidence, risk factors and outcome of AKI [22].

## Methods

### Study design

This was a cross-examination of a retrospective analysis of clinical data of patients who underwent elective or urgent major nonvascular abdominal surgery at the Department of Surgery II of Centro Hospitalar Lisboa Norte from January 2010 to February 2011. [22] The study was approved by the Ethical Committee at the Centro Hospitalar Lisboa Norte, EPE, in agreement with institutional guidelines. Informed consent was waived by the Ethical Committee due to the retrospective and non-interventional nature of the study.

### Participants

All patients aged 18 or older who underwent urgent or elective major nonvascular abdominal surgery admitted to the Post-Anesthesia Care Unit (PACU) of the Department of Surgery II of Centro Hospitalar Lisboa Norte from January 2010 to February 2011 were eligible for this study.

Major abdominal surgery was defined as intraperitoneal approach performed under general anesthesia, with a predictable length of hospital stay of at least two days. [23, 24] For patients with more than one surgery, only the first procedure was considered. In patients with multiple hospital admissions, only the first one was considered.

**Fig. 1:**
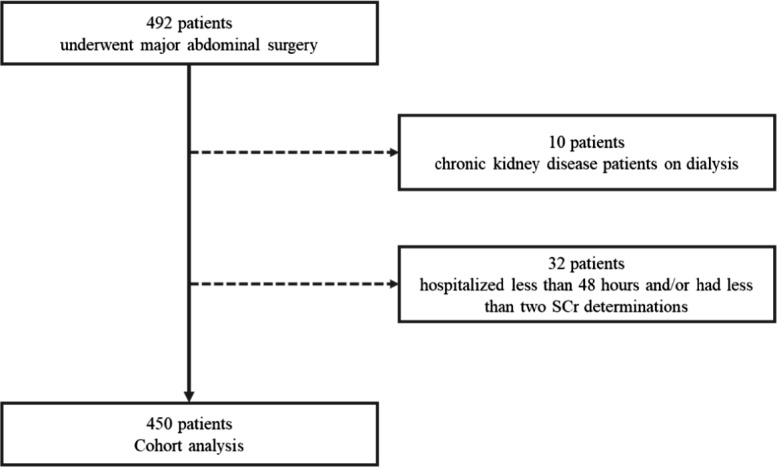
Flow-chart of patient selection

Exclusion criteria included: CKD patients already on renal replacement therapy; patients who underwent renal replacement therapy the week before surgery; patients who had less than two determinations of SCr during hospital stay, and patients who were discharged from hospital less than two days after the procedure. After analysis of the PACU patient admission register, 492 patients were selected as potentially eligible. Of these, 42 were excluded. [22] (Fig. 1).

### Variables

All variables were collected from electronic and hand-written patient clinical records, including intraoperative data recorded by the anaesthesiologist. All scores and formulas were calculated based on raw clinical data.

The analyzed variables included demographic characteristics (age, gender, and ethnicity), preoperative clinical characteristics, physical status according to the American Society of Anaesthesiologists (ASA) score [25], preoperative serum hemoglobin and SCr, nature of the procedure (elective or urgent), duration of anaesthesia, intraoperative blood pressure, use of fluids (colloids - hydroxyethyl starch, gelatin and albumin 5%; crystalloids - sodium chloride 0.9%, Ringer’s lactate and polyelectrolyte solution), intraoperative blood transfusions and use of vasoactive drugs, postoperative AKI and mortality.

Regarding preoperative clinical characteristics, the comorbidities registered were diabetes mellitus (diagnosed according to the American Diabetes Association criteria [26], hypertension (diagnosed according to the seventh report of the Joint National Committee [27], cardiovascular disease (including chronic heart failure, cardiac ischemic disease and history of transient ischemic attack or stroke), chronic obstructive pulmonary disease (COPD) including emphysema and chronic bronchitis, and malignancy. For cardiovascular disease and COPD, indication on clinical records of previous diagnosis was considered sufficient. To estimate the glomerular filtration rate (eGFR) the 4-variable Modification of Diet in Renal Disease formula was used [28].

Pertaining intraoperative variables, systolic and diastolic blood pressure (SBP and DBP, respectively) were recorded automatically every 5 min and intraoperative mean arterial pressure (MAP) was calculated as [(2xDBP) + SBP]/3. When available, invasive measurements were preferred to non-invasive ones. Intraoperative hypotension (IOH) was defined as intraoperative MAP inferior to 65 mmHg and the number of episodes of IOH was registered. Blood transfusions were done in patients with active bleeding or hemodynamically unstable or when the serum hemoglobin level was below the 7 to 8 g/dl range [29] or, in older patients and in patients with coronary artery disease, bellow 10 g/dl.

### Definitions

In this analysis, only AKI developing in the first 48 h after surgery was considered to be attributed to the surgical procedure. AKI was diagnosed using the Kidney Disease Improving Global Outcome (KDIGO) classification based on both serum creatinine (SCr) and urine output (UO) criteria, as an increase in serum creatinine (SCr) of 0.3 mg/dl within 48 h-periods, or an increase in SCr of 1.5 times baseline, which is known or within the prior 7 days, or a decrease in urinary output to less than 0.5 ml/kg/h for 6 h [30]. Pre-operative SCr was considered baseline SCr.

The neutrophil, lymphocyte and platelet counts were measured in the clinical laboratory of our hospital in the first 12 h after surgery. Maximal neutrophil counts and minimal lymphocyte and platelet counts were considered. Postoperative N/LP ratio was calculated as: (Neutrophil count × 100)/(Lymphocyte count x Platelet count).

### Outcomes

Development of AKI, in-hospital mortality and length of hospital and ICU stay were assessed.

### Statistical methods

Continuous variables were presented as the mean ± standard deviation and categorical variables as the total number and percentage of cases for each category. Normality of variables was assessed using the Kolmogorov-Smirnov test. After grouping participants according to the development of postoperative AKI, the variables of both groups were compared using Student’s t-test for normally distributed continuous variables and chi square test for categorical variables.

The discriminatory ability for N/LP ratio to predict AKI was determined using the receiver operating characteristic (ROC) curve. A cut-off value was defined as that with the highest validity.

Firstly, all variables underwent univariate analysis to determine statistically significant factors that may have contributed to AKI and mortality. Only variables which significantly differed between AKI and non-AKI groups were used in the multivariate analysis using the logistic regression method. An adjusted multivariate analysis to pre-operative, intra-operative and post-operative factors was conducted. Data were expressed as Odds ratios (OR) with 95% confidence intervals (95% CI). Sensitive analysis excluding septic patients and adjusting for pre-operative, intra-operative and post-operative factors were conducted.

Statistical significance was defined at a *p*-value (*p*) < 0.05. Analyses were performed with the statistical software package SPSS 21.0 for Windows.

## Results

### Participants

We focused on a cohort of 450 patients. Demographic, preoperative, intraoperative and postoperative patient variables and outcomes are described in Tables 1 and 2.

**Table 1:** Patient’s baseline characteristics and comparison according to the development of AKI

Characteristic	Baseline (*n* = 450)	KDIGO		
		No AKI (*n* = 349)	AKI (*n* = 101)	*p* value
Age (years) - mean ± SD	62 ± 16	60.6 ± 15.7	71.1 ± 13.2	< 0.0001
Gender (Male) – n (%)	227 (50.4)	168 (48.1%)	59 (58.4%)	0.069
Race (Caucasian) – n (%)	431 (95.8)	335 (96)	96 (95)	0.679
Co-morbidities – n (%)				
Hypertension	225 (50)	168 (48.1)	57 (56.4)	0.142
Diabetes	84 (18.7)	62 (17.8)	22 (21.8)	0.001
CVD	29 (6.4)	13 (3.7)	16 (15.8)	< 0.0001
COPD	25 (5.6)	13 (3.7)	12 (11.9)	< 0.0001
Cirrhosis	9 (2)	7 (2)	2 (1.9)	0.987
Neoplasia	190 (42.2)	135 (38.7)	55 (54.5)	0.005
SAPS II - n (%)	23 (17.4)	19.1 (10.9)	34.3 (21.3)	< 0.0001
Baseline SCr (mg/dl) - mean ± SD	1 ± 0.5	0.9 ± 0.4	1.2 ± 0.7	< 0.0001
Baseline eGFR (ml/min/1.73 m2) - mean ± SD	80.3 ± 1.1	92.5 ± 1.2	60.5 ± 1.3	< 0.0001
Hemoglobin (g/dl) - mean ± SD	11.1 ± 1.7	11.3 ± 1.7	10.5 ± 1.6	< 0.0001
Post-operative neutrophils - mean ± SD	9.2 ± 4.2	9.39 ± 4.35	8.61 ± 3.57	0.102
Post-operative lymphocytes - mean ± SD	1.2 ± 0.8	1.26 ± 0.90	0.97 ± 0.61	0.003
Post-operative platelets - mean ± SD	243.1 ± 105.2	245.95 ± 104.94	233.05 ± 106.2	0.279
Post-operative NL/P - mean ± SD	4.78 ± 4.64	4.33 ± 3.36	6.36 ± 7.34	< 0.001
Post-operative NL/P > 4.86– n (%)	154 (34.2)	113 (32.4)	54 (53.5)	< 0.001
Pre-operative sepsis – n (%)	70 (15.6)	39 (11.2)	31 (30.7)	< 0.001
Mechanical ventilation – n (%)	11 (2.4)	6 (1.7)	5 (4.9)	0.064
Vasopressors – n (%)	80 (17.7)	55 (15.8)	25 (24.8)	0.037
Fluid balance in the first 48 h (L) - mean ± SD	1.48 ± 1.43	1.40 ± 1.42	1.72 ± 1.44	0.046
LOS in hospital (days) - mean ± SD	12 ± 12.6	11.1 ± 11.3	15.2 ± 16.1	0.004
LOS in ICU (days) - mean ± SD	20 ± 4.4	3.6 ± 7.1	6.9 ± 11.7	< 0.0001
Hospital mortality – n (%)	29 (6.4)	8 (2.3)	21 (20.8)	< 0.0001

Abbreviations: *CVD* cardiovascular disease, *COPD* chronic obstructive pulmonary disease, *SCr* serum creatinine, *eGFR* estimated glomerular filtration rate, *NL/P* neutrophil-lymphocyte and platelet ratio, *SD* standard deviation; LOS length of stay; ICU intensive care unit

**Table 2:** Univariate and multivariate analysis of factors predictive of outcomes

	AKI				Mortality			
	Unadjusted OR (95% CI)	*P*-value	Adjusted OR (95% CI)	*P*-value	Unadjusted OR (95% CI)	*P*-value	Adjusted OR (95% CI)	*P*-value
Preoperative characteristics								
Age	1.05 (1.04–1.07)	< 0.0001	1.04 (1.0 2-1.06)	< 0.0001	1.1 (1.06–1.2)	< 0.0001	1.10 (1.03–1.17)	0.003
Male	1.5 (0.9–2.4)	0.07			1.1 (0.5–2.2)	0.887		
Caucasian	0.8 (0.3–2.3)	0.680			1.3 (1.2–9.7)	0.831		
Diabetes	0.046 (0.01–0.15)	0.363			0.01 (0.01–0.05)	0.839		
CVD	0.35 (0.2–0.5)	< 0.0001	0.23 (0.089–0.38)	0.002	0.115 (0.023–0.21)	0.14		
COPD	0.31 (0. 15-0.48)	< 0.0001	0.22 (0.07–0.37)	0.005	0.059 (0.001–0.158)	0.245		
Solid malignancy	1.9 (1. 2-2.9)	0.005	1.47 (0.85–2.54)	0.174	1.5 (0. 7-3.2)	0.287		
ASA physical status IV/V	2.5 (1. 2-4.9)	0.01	1.16 (0.49–2.78)	0.732	15.4 (6. 7-35.6)	< 0.0001	10.71 (2.90–39.54)	< 0.0001
Hemoglobin (g/dL)	0.8 (0. 7-0.9)	< 0.0001	0.97 (0.82–1.14)	0.675	0.63 (0. 5-0.8)	< 0.0001	0.73 (0.51–1.04)	0.077
Baseline SCr (mg/dL)	2.3 (1. 5-3.4)	< 0.0001	1.28 (0.78–2.12)	0.327	2.3 (1. 3-3.9)	0.003	0.56 (0. 24-1.30)	0.174
Preoperative sepsis	3.52 (2.06–6.03)	< 0.0001	1.30 (0.63–2.66)	0.480	19.9 (8. 37-47.43)	< 0.0001	5.23 (1.46–18.75)	0.011
Urgency surgery	0.3 (0. 8-2.1)	0.368			4.2 (1. 9-8.9)	< 0.0001	1.32 (0. 33-5.22)	0.693
Intraoperative characteristics								
Duration of anesthesia	1.02 (1-1.05)0.024	0.024	1.00 (1.00–1.00)	0.156	1.00 (0.997–1.004)	0.845		
Hypotension episodes	1.2 (1. 1-1.4)	0.001	1.10 (0.95–1.26)	0.203	1.46 (1. 27-1.69)	< 0.0001	1.52 (1. 16-1.98)	0.002
Crystalloids with colloids	3 (1. 8-5)	< 0.0001	1.80 (0.97–3.33)	0.062	6.05 (1.80–20.29)	0.004	2.61 (0.43–15.68)	0.296
Fluid balance	1.00 (1.00–1.001)	0.048			1.00 (1.00–1.001)	< 0.0001		
Erythrocytes (per unit)	3.7 (2. 4-5.8)	< 0.0001	2.27 (1.45–3.55)	< 0.0001	2.25 (1.49–3.40)	< 0.0001	1.22 (0.68–2.20)	0.508
Vasoactive drugs use	1.8 (1. 1-3)	0.039	1.10 (0.58–2.11)	0.769	5.87 (2.70–12.74)	< 0.0001	4.08 (1. 21-13.78)	0.023
Post-operative characteristics								
NL/P (per point increase)	1.1 (1.04–1.16)	0.001	1.05 (1.00–1.10)	0.048	1.04 (0.995–1.106)	0.078	0.95 (0.84–1.08)	0.463
NL/P > 4.86	2.4 (1.53–3.77)	< 0.0001	1.72 (1.04–2.87)	0.036	2.56 (1. 19-5.5)	0.178	1.16 (0. 38-3.5)	0.798
AKI					11.19 (4.78–27.18)	< 0.0001	3.56 (1.02–12.43)	0.046

In the first 48 h after surgery, 101 patients (22.4%) developed AKI. Patients with post-operative AKI were older (*p* < 0.001) and were more likely to have preexisting ischemic heart disease (*p* = 0.001), congestive heart failure (*p* = 0.008), cerebrovascular disease (*p* < 0.001), COPD (*p* < 0.001) and solid malignancies (*p* = 0.005) and to be ASA I*V*/V (*p* = 0.009). Also, these patients had lower preoperative hemoglobin (Hb) values (*p* < 0.001), higher preoperative SCr (*p* < 0.001) and preoperative sepsis (*p* < 0.001). Longer duration of anesthesia (*p* = 0.021), intraoperative hypotension episodes (*p* < 0.001), intraoperative colloids and crystalloids as compared with crystalloids without colloids (*p* < 0.001), intraoperative erythrocytes transfusions (*p* < 0.001), and intraoperative vasoactive drugs (*p* = 0.037) were also associated with AKI.

### Post-operative N/LP ratio and AKI

Patients with higher N/LP ratio had an increased risk of developing postoperative AKI (6.36 ± 7.34 vs 4.33 ± 3.36, *p* < 0.001; unadjusted OR 1.1 (95% CI 1.04–1.16), *p* = 0.001; adjusted OR 1.05 (95% CI 1.00–1.10), *p* = 0.048).

In a multivariate analysis only older age (*p* < 0.001), CVD (*p* = 0.002), COPD (*p* = 0.005), NL/P ratio (*p* = 0.048) and intraoperative erythrocytes transfusions (*p* < 0.001) were associated with AKI. After a sensitive analysis in which septic patients were excluded, a higher NL/P was still associated with AKI development (6.29 ± 8.1 vs 4.14 ± 3.01, *p* < 0.001; unadjusted OR 1.11 (95% CI 1.036–1.189), *p* < 0.001; adjusted OR 1.078 (95% CI 1.01–1.15), *p* = 0.025). An adjusted multivariate analysis to pre-operative, intra-operative and post-operative factors was conducted, in which a higher N/LP ratio remained as an independent predictor of increased risk of developing postoperative AKI (adjusted OR 1.012 (95% CI 1.003–1.021), *p* = 0.007).

**Fig. 2:**
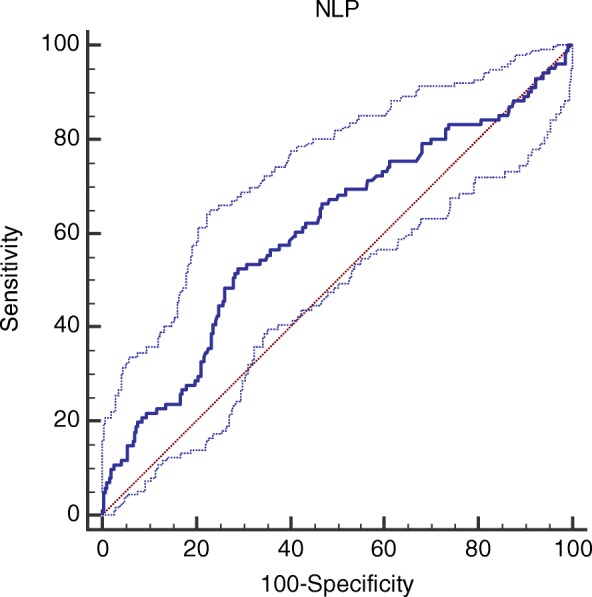
AUC of the risk model for the prediction of AKI with NL/P ratio

A ROC curve was produced to assess the discriminative ability of N/LP ratio for AKI. The AUC for AKI prediction was of 0.606, 95% CI (0.559–0.651). (Fig. 2) The optimal cutoff was assessed to be > 4.86, which has a sensitivity of 52.5% and specificity of 71.1% with a positive predictive value of 1.81 and negative predictive value of 0.67. In a multivariate analysis, NL*P* > 4.86 was associated with an increased risk of postoperative AKI (adjusted OR 1.72 (95% CI 1.04–2.87), *p* = 0.036).

### Post-operative N/LP ratio and mortality

In this cohort 29 patients died, and AKI was an independent predictor of mortality (20.8 vs 2.3%, *p* < 0.0001; unadjusted OR 11.2, 95% CI 4. 8-26.2, *p* < 0.0001; adjusted OR 3.56, 95% CI 1.02–12.43, *p* = 0.046). In a multivariate analysis higher N/LP ratio was not associated with increased in-hospital mortality.

## Discussion

In this retrospective cohort, we demonstrated that a higher postoperative N/LP ratio was independently associated with AKI after major abdominal surgery, whereas there was no association with in-hospital mortality.

In past years, many studies have revealed the important role of inflammation in the pathogenesis of AKI, and in particular in postoperative AKI [8–10].

Release of endotoxin load from gut ischemia, impaired visceral perfusion, and portal endotoxemia activate a pro-inflammatory response during abdominal surgery. [11] This results in endothelial injury and consequently vasoconstriction, microvascular sludging, and congestion with leukocytes. [31] Additionally, the postischemia or reperfusion period induces further tubular damage produced by reactive oxygen species and tissue inflammation. [10, 11, 31] The immune activation following AKI results in systemic inflammatory changes. [32].

Platelets have a central role in maintaining hemostasis and coagulation. [33] Recent data supports the present role of platelets in inflammation. [33] Their interaction with neutrophils, monocytes, and lymphocytes modulates both innate and adaptive immune responses. [34] Platelets adhere to damaged endothelium and recruit leukocytes to sites of injury. [35] Moreover, it has been shown that the microvascular sludging with leukocytes and activated platelets is critical in the pathogenesis of postoperative AKI. [31, 36].

Postoperative thrombocytopenia might result from this microvascular sludging and platelet consumption. In cardiac surgery the association of postoperative thrombocytopenia and AKI has been demonstrated. [36] In fact, the extent of the platelet count decrease was associated with the severity of AKI and hospital mortality. [36] Thrombocytopenia has also been associated with AKI and mortality in non-surgical settings. [37, 38] Nonetheless, thrombocytopenia is also a marker for critically ill patients and may reflect an underlying disease. [33].

The N/L ratio has already been established as an important marker for inflammation in AKI. [13, 18–20] In order to combine the predictive ability of the N/L ratio and platelet count, Koo et al. developed the N/LP ratio in the setting of postoperative AKI after cardiovascular surgery. [21] In a multivariate analysis, the ratio was independently associated with AKI differently than separate N/L ratio and platelet nadir. [21] Their data also reports an association between the N/LP ratio and short and long-term mortality. [21].

In our cohort of AKI after major abdominal surgery, N/LP ratio was independently associated with AKI, however no association with mortality was demonstrated. These results may reflect the small sample of our cohort and further study is necessary to apply this ratio as a mortality predictor.

The main strength of our study is that this is the first to examine the association between N/LP and AKI after major abdominal surgery, confirming the impact of inflammation in this setting. Also, some important variables were accounted for in our study. Transfusion amount has been reported as a predictor for postoperative AKI [22], nevertheless, the association of the ratio with AKI remained significant after adjustment for these variables. We used a marker of inflammation and accounted for septic patients prior to the surgery, in order to assess only the inflammation associated to surgery related AKI, and the N/LP ratio remained a significant predictor of AKI.

However, there are some important limitations. Firstly, the single-center nature of our study which limits generalizability. Second, the retrospective design with a moderately small cohort of patients which may have overlooked some potential confounders with prognostic importance. Finally, the predictive value of our ROC curve is modest, nevertheless indicative of a significant association of the variables, meaning that further validation of this ratio is required.

## Conclusion

In conclusion, we confirmed that the postoperative N/LP ratio was independently associated with AKI after major abdominal surgery, although there was no association with in-hospital mortality in our cohort. The assessment of this ratio is straightforward from routine blood analysis in postoperative patients and can prove useful in identifying patients at risk for AKI.
